# Supplementary material for: CrowdGO: Machine learning and semantic similarity guided consensus Gene Ontology annotation
Source: PLoS Comput Biol. 2022 May 13;18(5):e1010075. doi: 10.1371/journal.pcbi.1010075 (PMC9132264; doi:10.1371/journal.pcbi.1010075)
Supplement: S1 Text — Table A: Checklist for reporting and evaluating machine learning models. Table B: Comparisons of CrowdGO annotations with baseline homology-based annotations. Fig A: Distributions of GO terms per protein for the V162-V198 training and testing datasets and the CAFA3 benchmarking dataset. Distributions are shown as density plots (A-C) and as empirical cumulative distribution function (ECDF) plots (D-E) for the Molecular Function (MFO), Biological Process (BPO), and Cellular Component (CCO) ontologies. The total number of proteins in each dataset are shown on the plots, along with results from Wilcoxon (Mann-Whitney) tests for each pair of datasets for A-C, and with results of Kolmogorov-Smirnov tests for each pair of datasets for D-F. Analysis, plotting, and significance testing all performed using R 3.6.1. The V162-V198 training and testing datasets show no significant differences in their distributions of GO terms per protein. Compared with the CAFA3 dataset, they are both significantly lower for MFO, significantly higher for BPO, and not significantly different for CCO. Fig B: Distributions of information contents for GO terms from the V162-V198 training and testing datasets and the CAFA3 benchmarking dataset. Distributions are shown as density plots (A-C) and as empirical cumulative distribution function (ECDF) plots (D-E) for the Molecular Function (MFO), Biological Process (BPO), and Cellular Component (CCO) ontologies. The total number of gene-term annotations in each dataset are shown on the plots, along with results from Wilcoxon (Mann-Whitney) tests for each pair of datasets for A-C, and with results of Kolmogorov-Smirnov tests for each pair of datasets for D-F. Analysis, plotting, and significance testing all performed using R 3.6.1. The V162-V198 training and testing datasets show no significant differences in their distributions of information contents (ICs). Compared with the CAFA3 dataset, their medians are not significantly different for MFO but their [file pcbi.1010075.s001.docx]

**CrowdGO: machine learning and semantic similarity guided consensus Gene Ontology annotation**

Maarten J.M.F. Reijnders^1^, Robert M. Waterhouse^1^

^1^ Department of Ecology and Evolution, University of Lausanne, and Swiss Institute of Bioinformatics, 1015 Lausanne, Switzerland

***Supplementary Information***

Contents

[Supplementary Materials and Methods 2](#_Toc101346560)

[Proteins and gene ontology annotations 2](#_Toc101346561)

[Running input predictors and CrowdGO 3](#_Toc101346562)

[Performance evaluation metrics 4](#_Toc101346563)

[Assessing impact of CrowdGO re-evaluations 5](#_Toc101346564)

[Annotating model and non-model species 5](#_Toc101346565)

[CrowdGO software workflow 7](#_Toc101346566)

[Supplementary Tables and Figures 10](#_Toc101346567)

[Table A. Checklist for reporting and evaluating machine learning models 10](#_Toc101346568)

[Table B. Comparisons of CrowdGO annotations with baseline homology-based annotations. 12](#_Toc101346569)

[Fig A: Distributions of GO terms per protein for the V162-V198 training and testing datasets and the CAFA3 benchmarking dataset. 13](#_Toc101346570)

[Fig B: Distributions of information contents for GO terms from the V162-V198 training and testing datasets and the CAFA3 benchmarking dataset. 13](#_Toc101346571)

[Fig C: Sequence identity distributions of the V162-V198 training and testing datasets. 14](#_Toc101346572)

[Fig D: CrowdGO applied to functionally annotate the complete proteomes of taxonomically diverse species. 14](#_Toc101346573)

[Supplementary References 19](#_Toc101346574)

# Supplementary Materials and Methods

## Proteins and gene ontology annotations

The V162-V198 training, testing, and CAFA3 benchmarking GO annotation sets were built following best practices for defining datasets for evaluating machine learning models (Table A) and CAFA3 benchmark generation guidelines.

***The V162-V198 dataset***: The set of annotations for building the AdaBoost models was derived from all proteins added to the GOA UniProt database from version 162 (17-01-2018) to version 198 (17-06-2020). Only annotations for proteins from the manually annotated SwissProt subset of UniProt, and with evidence codes EXP, IDA, IPI, IMP, IGI, IEP, TAS, or IC were retained, and proteins included in the CAFA3 benchmarking datasets were removed. If the only MFO term for a protein was ‘GO:0005515’ (protein binding), this was removed from both the training and test sets (326 out of 967 proteins), in line with CAFA3 benchmark generation guidelines. Finally, all parent GO terms from the GO DAG (release 2016-11-12) were propagated onto each of the annotations using the ‘is_a’ and ‘part_of’ relationships. This resulted in 661 proteins annotated with 4,477 MFO terms (397 unique, 3.3 % of MFO), 1,988 with 61,043 BPO terms (2,800 unique, 9% of BPO), and 2,466 with 33,564 CCO terms (407 unique, 9.3% of CCO). This complete V162-V198 dataset used to train the AdaBoost models is provided with CrowdGO. In order to compare CrowdGO results with annotations from individual input predictors, this dataset was split by randomly assigning 50% of proteins into a V162-V198 training subset and a V162-V198 testing subset.

***The CAFA3 benchmarking dataset***: CAFA benchmarking datasets of proteins and their GO terms rely on annotation growth over time and are generated by selecting all proteins that have gained experimental annotations during a set period. To assess the performance of CrowdGO results compared with annotations from the baseline and top-performing CAFA3 predictors, the CAFA3 no-knowledge (type1) benchmarking dataset was used, which consists exclusively of proteins without any prior annotations. This CAFA3 benchmarking dataset consists of new GO term annotations added to the GOA UniProt database from 13-02-2017 to 15-11-2017 as detailed in [1].

The average numbers of leaf and parent-propagated GO terms per protein are generally similar across the three datasets, apart from slightly higher averages for CAFA3 MFO and nearly double the leaf annotations per protein for BPO in the V162-V198 datasets. Terms per protein and term ICs are also well-matched between the training and testing datasets, showing no significant differences in their distributions (Supplementary Figures S1 and S2). Sequence identity distributions of the V162-V198 training and testing datasets against the CAFA3 benchmarking dataset, and the V162-V198 training against the V162-V198 testing datasets show no substantial deviations or biases towards elevated proportions of highly similar sequences (Figure S3), therefore no sequence-similarity-based redundancy-reducing was performed.

***Model and non-model species proteomes***: CrowdGO was applied to complete proteomes (all protein-coding genes in the genome) for 12 model and non-model species to compare the results with their existing annotations. These, together with their SwissProt (where available) and TrEMBL annotations, were retrieved from the UniProt proteomes (downloaded 18-05-2020) for: *Anopheles gambiae* mosquito (UP000007062); *Arabidopsis thaliana* mouse-ear cress (UP000006548); *Candidatus Thorarchaeota archaeon* SMTZ1-45 (UP000070149); *Chlamydomonas reinhardtii* green alga (UP000006906); *Drosophila melanogaster* fruit fly (UP000000803) *Escherichia coli* K12 Gram-negative bacterium (UP000000625); *Homo sapiens* human (UP000005640); *Pan troglodytes* chimpanzee (UP000002277); *Pseudomonas putida* Gram-negative bacterium (UP000250299); *Saccharomyces cerevisiae* yeast (UP000002311); *Solanum lycopersicum* tomato (UP000004994); and *Yarrowia lipolytica* yeast (UP000256601).

## Running input predictors and CrowdGO

CrowdGO performance was assessed by comparing the consensus results with four input annotation sets from running: DeepGOPlus [2], Wei2GO [3], InterProScan [4], and FunFams [5], on the V162-V198 testing dataset. Importantly, these methods were applied using their corresponding datasets and models that pre-date the annotations in the testing dataset. DeepGOPlus (GitHub 13-09-2020, using the DeepGOPlus 2016 dataset) was run with default parameters. DeepGOPlus and Wei2GO require DIAMOND [6] for protein sequence searches, and were run with version v0.9.11.112. Wei2GO and FunFams require HMMScan and HMMSearch functions from HMMER [7], and were run using HMMER version 3.3.1. Wei2GO v1.0 was run with default parameters using sequences and annotations from UniProt release 2016-11 and profiles from Pfam-A [8] version 30 (2016-05), i.e. with datasets that predate the sequences in the V162-V198 dataset to ensure no overlaps. InterProScan version 5.21.60 (2016-11) was run with default parameters and the --goterms option to obtain GO term annotations, using all default member databases as well as PANTHER protein families from version 10 [9], i.e. families that predate the sequences in the V162-V198 dataset. FunFams (cath-tools-genomescan GitHub 17-12-2019) was run with default parameters with CATH database version 4.1 [10], which was based on Protein Data Bank (PDB) release 01-01-2015. InterProScan and FunFams do not provide probability scores for their predictions. For FunFams these were computed as detailed in [11], briefly: annotation frequency or normalised GO term occurrence count takes into account the likelihood of co-occurring with protein domains with other functions, so that each FunFams family is associated with a term-specific probability value. Probability scores for assignments from different InterProScan member databases are not directly comparable, so all InterProScan predictions were set to a score of 1. Finally, an AdaBoost model was built using results from annotating the V162-V198 training dataset using DeepGOPlus, Wei2GO, InterProScan, and FunFams. This AdaBoost model was then used to re-evaluate each gene-term annotation score and produce the new consensus dataset of annotations for the V162-V198 testing dataset. Performance was also compared with CAFA3 predictors, using the pre-trained AdaBoost models provided with CrowdGO to produce consensus annotations from results obtained by running DeepGOPlus, Wei2GO, InterProScan, and FunFams on the CAFA3 benchmarking dataset.

## Performance evaluation metrics

Performance was assessed using the V162-V198 training and testing datasets to compare with the four input methods, and using the CAFA3 no-knowledge benchmarking dataset to compare with CAFA3 predictors. For all evaluation metrics, threshold steps of probability scores of 0.01 were used for outputs of each predictor (except for InterProScan due to uniform probability score of 1), with Wei2GO probability scores first scaled from zero to one. The F1-score summarises performance as the harmonic mean between precision and recall, with *F_max_* as the highest F1-score across all thresholds [12]. Considering GO term ICs is also important, as annotating general terms is less useful than predicting more specific functions. The *S_min_* score measures the minimum semantic distance between the predicted and real annotations across all thresholds [13]. These standard benchmarking methods were used to define the evaluation metrics for precision, recall, *F_max_*, *S_min_*, and area under the precision-recall curve (AUPR) calculated as follows (note, equations 1 and 2 are presented in the main text):

${pr}_{i}(t)= \frac{\sum_{f} I(f\in P_{i}(t)\wedge f\in T_{i})}{\sum_{f} I(f\in P_{i}(t))}$ (3)

${rc}_{i}(t) = \frac{\sum_{f} I(f\in P_{i}(t)\wedge f\in T_{i})}{\sum_{f} I(f\in T_{i})}$ (4)

$AvgPr\left( t \right)= \frac{1}{m(t)} \cdot\sum_{i=1}^{m(t)} {pr}_{i}(t)$ (5)

$AvgRc\left( t \right) = \frac{1}{n} \cdot\sum_{i=1}^{n} {rc}_{i}(t)$ (6)

Where for each threshold (*t*) the precision (*pr*) and recall (*rc*) are calculated per protein (*i*), $f$ is a GO term, *T_i_* is the set of true terms annotated to protein _i_, and *P_i_* the set of predicted terms annotated to protein _i_, *m*(*t*) is the number of proteins for which at least one term is predicted above the threshold, *n* is the total number of proteins, and *I* is an identity function which returns 1 if True and 0 if False. The maximum F1-score is then calculated, with thresholds ranging from 0 to 1 in steps of 0.01.

$F_{max}=\max_{t} \{\frac{2\cdot AvgPr(t)\cdot AvgRc(t)}{AvgPr\left( t \right)+AvgRc(t)}\}$ (7)

*S_min_* is computed per protein based on the remaining uncertainty (*ru*), which is the IC of all GO terms *g* that comprise the subset of true terms annotated to the protein that were not predicted (False Negatives), and the missing information (*mi*), which is the IC of all terms *g* that comprise the subset of predicted terms that are not in the set of true terms annotated to the protein (False Positives).

$S_{min}=\min_{t} \sqrt{{ru(t)}^{2}+{mi(t)}^{2}}$ (8)

Where *ru* and *mi* are calculated as:

$ru\left( t \right)=\frac{1}{n}\sum_{i=1}^{n} \sum_{c\in Ti-Pi(t)} IC(c)$ (9)

$mi\left( t \right)=\frac{1}{n}\sum_{i=1}^{n} \sum_{c\in Pi\left( t \right)-Ti} IC(c)$ (10)

## Assessing impact of CrowdGO re-evaluations

To examine the effects of CrowdGO re-evaluations of the input annotation sets, correct and incorrect classifications of true and false positive and negative annotations were enumerated by comparing annotations before and after applying CrowdGO. Each gene-term annotation from the predictions of all input methods on the V162-V198 testing set was labelled true positive (tp), false positive (fp), true negative (tn), or false negative (fn). All thresholds used for assigning positive or negative labels were based on the *F_max_* scores that each input method achieved on the V162-V198 testing dataset. Confident CrowdGO consensus annotations (scores of 0.5 or higher) were labelled positives and the rejected annotations were labelled negatives. The total numbers of correct (fn->tp; fp->tn) and incorrect (tp->fn; tn->fp) reclassifications, and of classification affirmations that were correct (tp->tp; tn->tn) or incorrect (fn->fn; fp->fp), were visualised using Sankey plots for each ontology.

## Annotating model and non-model species

CrowdGO was applied to annotate the proteomes of 12 species using default parameters and the provided CrowdGOFull AdaBoost model. FunFams was run using default parameters with version funfam-hmm3-v4_3_0.lib (based on PDB release 01-07-2019); InterProScan was run using default parameters and the --goterms option with version 5.45-80.0 (18-06-2020); Wei2GO was run using default parameters with UniProt release 06-2020 and Pfam version 33.0 (18-03-2020); and DeepGOPlus was run with default parameters using the GitHub repository downloaded on 01-06-2020. Consensus CrowdGO results were compared with existing annotations for the 12 species from SwissProt (where available) and TrEMBL databases from UniProt. All annotations from UniProt were included and compared to confident CrowdGO annotations (scores of 0.5 or higher). For both UniProt and CrowdGO datasets, all parent GO terms were propagated onto each gene-term annotation using the GO DAG (obo version 23-03-2020). To quantify the breadth of terms annotated to each protein, counts of terms per protein were calculated by summing all parent terms that are part of the GO slim ‘Generic GO Subset’ (downloaded 10-10-2020). GO term ICs computed as defined in equation (1) were used to quantify annotation depth by summing the IC of all terms (including parent terms) annotated to each protein. These measures of annotation breadth (counts of GO Slim terms per protein) and depth (summed IC of terms and their parents) were used to compare CrowdGO results with existing annotations for each species.

Compute times will vary according to the resources available and the flexibility of each input predictor to run assessments in parallel. The task that generally takes the longest is running InterProScan, e.g. annotating the human proteome using 8 CPUs, the default, took approximately 24 hours. Using 1 CPU, FunFams completed in 4 hours, Wei2GO produced annotations in 4 hours and DeepGOPlus annotated the human proteome in 30 minutes. In contrast, the final step of combining all the annotations to produce the consensus with CrowdGO took only 30 minutes.

To compare CrowdGO annotations with baseline homology-based GO annotations, an approach similar to the CAFA3 baseline was applied to the 12 proteomes. The proteins were used as queries to search the UniProt database of proteins using DIAMOND [6] with default parameters. For every query protein the top UniProt hit (lowest e-value) was extracted and GO term annotations were transferred from the hit to the query based on the GOA database. The CrowdGO annotations were then compared to the baseline homology-based approach, for each of the three ontologies, by counting the total numbers of annotations, the numbers in common to both approaches, the numbers unique to each approach, and the annotations for each with a depth of more than 5 for Biological Process and Molecular Function terms, and a depth of more than 3 for Cellular Component terms. GO terms counted are exclusively leaf terms, i.e. terms for which no child terms are annotated. In calculating the unique terms for each approach, its leaf annotations were compared to the full annotations up the GO DAG of the other approach. All databases used were the same as for the CrowdGO annotations used in the annotation of the proteomes described in Figure 3.

## CrowdGO software workflow

**Overview**

Steps 1 & 2: providing CrowdGO with the annotations from the input predictors to then be able to proceed with either producing consensus annotations or training a new model.

Steps 3-6: defining GO term relations, namespaces, and summary statistics based on the selected GO graph and GOA database.

Step 7: collecting all similar GO terms per protein based on the computed summary statistics and the semantic similarity thresholds.

Step 8: re-evaluating scores for all predictions (when producing consensus annotations) or building a new AdaBoost model (when creating a new model).

Step 1: read the user input arguments

**Function name:** readArguments()

**Function purpose:** reads the user-given CrowdGO input file, output directory, and model file (only when predicting GO terms)

CrowdGO input predictions are given in a tabular format using five columns for each prediction when training a model, and four columns for each prediction when predicting GO terms. Each row represents one prediction from one predictor: (1) name of one of the input predictors used in the trained model, (2) protein accession, (3) predicted GO term, (4) associated prediction score, (5) True or False label (only when training a model).

The output folder will contain the CrowdGO predictions and associated provenance when predicting GO terms, and will contain the trained model as a python pickled file and associated provenance when training a new model.

A model file is only given when predicting GO terms. Either one of the pre-trained models can be used, or a new model can be trained. It is highly recommended to use a model matching the ontology to be predicted, i.e. ‘bp.pkl’ for biological process terms, ‘mf.pkl’ for molecular function terms, and ‘cc.pkl’ for cellular component terms.

Step 2: read the input files

**Function name:** readInput()

**Function purpose:** reads the CrowdGO input files for training or predicting

This function reads the given input predictions from different predictors into a dictionary ‘predictionDictionary’.

Step 3: calculate GO relations according to the DAG

**Function name:** goSlim()

**Function purpose:** Creates several dictionaries containing direct and indirect GO parent and GO child relations for each GO term.

The data are parsed from the ‘goParents.tab’ and ‘goChildren.tab’ files in the CrowdGO data folder. A dictionary is made containing all direct and indirect parents of each GO term, a separate dictionary for all child terms of each GO term, and a dictionary containing all parent and child terms for each GO term. These dictionaries are later used to calculate similarities between GO terms predicted for a protein between the different input methods.

Step 4: parse the number of occurrences of each GO term in the GOA database

**Function name:** getGoCounts()

**Function purpose:** For each GO term its total occurrences in the GOA database are stored in a dictionary.

These data are parsed from the ‘goCounts.tab’ file in the CrowdGO data folder. A dictionary containing the counts for each GO term is returned, as well as a sum of the total GO count of the GOA database. These counts are later used to calculate the semantic similarity between GO terms for the same protein between different predictors.

Step 5: parse the namespace belonging to each GO term

**Function name:** getNameSpaces()

**Function purpose:** for each GO term its namespace, e.g. ‘biological_process’, is stored in a dictionary.

These data are parsed from the ‘nameSpaces.tab’ file in the CrowdGO data folder. GO namespaces are used to see if two GO terms are potentially related, and to calculate similarities between GO terms.

Step 6: calculate total GO terms belonging to a namespace

**Function name:**  namespaceGOCount()

**Function purpose:** store the total occurrences of all GO terms belonging to a specific namespace in a dictionary.

These data are parsed from a dictionary of GO counts calculated in step 4, and the dictionary of namespaces parsed in step 5. These total counts for a namespace are later used in the calculation of similarities between GO terms.

Step 7: create a dictionary of similar GO terms predicted between methods for each protein

**Function name:** createClusters()

**Function purpose:** for every predicted GO term, find similar GO terms predicted by different methods and store this information in a dictionary.

For each row in the input file, i.e. for each prediction by each predictor, finds the parent GO terms from the other predictors that are most similar. Only GO terms with a semantic similarity of 0.5 or higher are considered. This results in each prediction containing the original GO term, as well as potentially the GO term from each other predictor that is the most similar. Additional information that is stored: the information content for each GO term, the prediction score for each GO term as given by the original prediction, the semantic similarity between each GO term from each different method, and the total amount of different input predictors that predicted a similar GO term. When training a model, and additional ‘True’ or ‘False’ label is stored as a ‘1’ or ‘0’ respectively, to indicate whether the GO term prediction is correct.

Step 8: train a model or predict GO terms using a model

**Function name:** model()

**Function purpose:**  Either trains an AdaBoost model given the data created in step 7, or use an existing AdaBoost model to predict GO terms.

The features and labels (when applicable) are read from the files created in step 7. Output is written to the user specified output folder, with the model file and provenance when training a model, and the predictions and provenance when predicting GO terms.

The features and label files are used directly as an input to the AdaBoost model without scaling or normalizing, as AdaBoost does not benefit from this. AdaBoost is used with the following parameters: base_estimator=DecisionTreeClassifier, max_depth=5, algorithm=’SAMME, learning_rate=1.0, n_estimators=100,000. AdaBoost scores are calibrated using SKLearn’s CalibratedClassifierCV using the following parameters: cv=prefit, method=sigmoid,

When using ‘CrowdGO_train.py’ the model file is written to the output folder as ‘model.pkl’, and when using CrowdGO.py’ the predictions are written to the output folder as ‘crowdgo.tab’ and ‘crowdgo_raw.csv’ as the direct raw output of ADABoost for each input row.

# Supplementary Tables and Figures

### Table A. Checklist for reporting and evaluating machine learning models.

Based on *Best practices in machine learning for chemistry*, Artrith et al. Nature Chemistry vol. 13, pg 505–508, 2021, https://doi.org/10.1038/s41557-021-00716-z

| **1. Data sources** | |
| --- | --- |
| 1a. Are all data sources listed and publicly available? | All proteins used for the evaluation of CrowdGO in Figure 1, Figure 2, and Table 2 are available in UniProt and the GOA database.  All proteins used for the evaluation of Table 3 are taken from the CAFA3 challenge data set found at, amongst others, <https://github.com/ashleyzhou972/CAFA_assessment_tool>  All proteins used for the evaluation of Figure 3 are taken from UniProt.  Details are listed under the ‘Proteins and gene ontology annotations’ subsection of the ‘Materials and Methods’ section. |
| 1b. If using an external database, is an access date or version number provided? | CrowdGO uses several external databases. All versions used for the evaluation in Figure 1, Figure 2, Table 2, and Table 3 are found under the ‘Running input predictors and CrowdGO’ subsection of the ‘Materials and Methods’ section.  Additionally, Figure 3 uses the trained models on the data listed above, but uses different data for predicting GO terms. These can be found under the ‘Annotating model and non-model species’ subsection of the ‘Materials and Methods’ section. |
| 1c. Are any potential biases in the source dataset reported and/or mitigated? | All data used for the training and evaluation of CrowdGO, including its input predictors as part of its pipeline, are from before the data used for evaluation and thus no bias is introduced. The exception is the data used for Figure 3, but because this figure doesn’t evaluate against a test set and instead uses a subjective evaluation bias is no factor here. |
| **2. Data cleaning** | |
| 2a. Are the data cleaning steps clearly and fully described, either in text or as a code pipeline? | Most data cleaning steps as part of the CrowdGO pipeline used for the evaluation in this paper are described on the gitlab wiki <https://gitlab.com/mreijnders/CrowdGO>.  Additionally the training and test set is cleaned as described in the ‘Proteins and gene ontology annotations’ subsection of the ‘Materials and Methods’ section. |
| 2b. Is an evaluation of the amount of removed source data presented? | A report of the numbers of proteins removed from the training and test set is described in the ‘Proteins and gene ontology annotations’ subsection of the ‘Materials and Methods’ section. |
| 2c. Are instances of combining data from multiple sources clearly identified, and potential issues mitigated? | The paper describes a methodology on combining predictions from different sources and is elaborately described throughout. |
| **3. Data representations** | |
| 3a. Are methods for representing data as features or descriptors clearly articulated, ideally with software implementations? | Features used and a description of how the features are gathered are described under the ‘The CrowdGO Algorithm’ subsection of the ‘Materials and Methods’ section. The gathering of these features is available as part of the CrowdGO pipeline and software package. |
| 3b. Are comparisons against standard feature sets provided? | Not applicable |
| **4. Model choice** | |
| 4a. Is a software implementation of the model provided such that it can be trained and tested with new data? | All models described in the paper are available at <https://gitlab.com/mreijnders/CrowdGO> |
| 4b. Are baseline comparisons to simple/trivial models (for example, 1-nearest neighbour, random forest, most frequent class) provided? | CrowdGO is a predictor that combines existing predictors to improve predictions. These input predictors are thus provided as baselines to demonstrate CrowdGO does indeed improve over these predictors. |
| 4c. Are baseline comparisons to current state-of-the-art provided? | The CAFA3 top performers are used to compare against CrowdGO on the CAFA3 challenge set.  Additionally, several of the methods used as an input to CrowdGO, which are used as baseline comparisons as described in section 4b, are considered state-of-the-art. |
| **5. Model training and evaluation** | |
| 5a. Does the model clearly split data into different sets for training (model selection), validation (hyperparameter optimization), and testing (final evaluation)? | No hyperparameter optimization is performed.  The training and test set are split 50/50, based on proteins, as described in the ‘Proteins and gene ontology annotations’ subsection of the ‘Materials and Methods’ section. Additionally, the CAFA3 challenge data is used for testing, therefore these proteins are not used as part of the training set.  Table 1 provides an overview of all data sets used for training and testing. |
| 5b. Is the method of data split (data splitting (for example, random, cluster- or time-based splitting, forward cross-validation) clearly stated? Does it mimic anticipated real-world application? | Data splitting for the training and test set is described in the ‘Proteins and gene ontology annotations’ subsection of the ‘Materials and Methods’ section.  Otherwise not applicable. |
| 5c. Does the data splitting procedure avoid data leakage (for example, is the same composition present in the training and test sets)? | There is no overlap between the training and test set, as is described in the ‘Proteins and gene ontology annotations’ subsection of the ‘Materials and Methods’ section. |
| **6. Code and reproducibility** | |
| 6a. Is the code or workflow available in a public repository? | Yes: <https://gitlab.com/mreijnders/CrowdGO> |
| 6b. Are scripts to reproduce the findings in the paper provided? | Yes: <https://gitlab.com/mreijnders/CrowdGO> under the ‘supplementary’ section. |

### Table B. Comparisons of CrowdGO annotations with baseline homology-based annotations.

Total: all annotations; Depth > N: terms more than N levels from the root term (N=5 for MFO and BPO, N=3 for CCO).

| **Annotation Comparison** | | **Molecular**  **Function** | **Biological**  **Process** | **Cellular**  **Component** |
| --- | --- | --- | --- | --- |
| **Total**  **Annotations** | CrowdGO | 3,303,274 | 4,300,260 | 1,967,941 |
|  | Baseline | 2,785,042 | 4,332,189 | 1,426,219 |
|  | Difference:  CrowdGO-Baseline | 518,232 | -31,929 | 541,722 |
|  | In Common | 1,744,249 | 1,896,949 | 760,500 |
|  | Unique to  CrowdGO | 1,559,025 | 2,403,311 | 1,207,441 |
|  | Unique to  Baseline | 1,040,793 | 2,435,240 | 665,719 |
| **Annotations**  **Depth > N** | CrowdGO | 1,362,397 | 1,970,222 | 497,654 |
|  | Baseline | 1,012,183 | 1,997,852 | 436,805 |
|  | Difference:  CrowdGO-Baseline | 350,214 | -27,630 | 60,849 |

**Supplementary Figures A, B, C, and D.**

### Fig A: Distributions of GO terms per protein for the V162-V198 training and testing datasets and the CAFA3 benchmarking dataset.

Distributions are shown as density plots (A-C) and as empirical cumulative distribution function (ECDF) plots (D-E) for the Molecular Function (MFO), Biological Process (BPO), and Cellular Component (CCO) ontologies. The total number of proteins in each dataset are shown on the plots, along with results from Wilcoxon (Mann-Whitney) tests for each pair of datasets for A-C, and with results of Kolmogorov-Smirnov tests for each pair of datasets for D-F. Analysis, plotting, and significance testing all performed using R 3.6.1. The V162-V198 training and testing datasets show no significant differences in their distributions of GO terms per protein. Compared with the CAFA3 dataset, they are both significantly lower for MFO, significantly higher for BPO, and not significantly different for CCO.

### Fig B: Distributions of information contents for GO terms from the V162-V198 training and testing datasets and the CAFA3 benchmarking dataset.

Distributions are shown as density plots (A-C) and as empirical cumulative distribution function (ECDF) plots (D-E) for the Molecular Function (MFO), Biological Process (BPO), and Cellular Component (CCO) ontologies. The total number of gene-term annotations in each dataset are shown on the plots, along with results from Wilcoxon (Mann-Whitney) tests for each pair of datasets for A-C, and with results of Kolmogorov-Smirnov tests for each pair of datasets for D-F. Analysis, plotting, and significance testing all performed using R 3.6.1. The V162-V198 training and testing datasets show no significant differences in their distributions of information contents (ICs). Compared with the CAFA3 dataset, their medians are not significantly different for MFO but their distributions are significantly flatter (the peak in the CAFA3 dataset arises from GO:0042802 ‘identical protein binding’ with an IC of 6.5 annotated to 61 proteins, the most proteins for any term). Their ICs are significantly higher than the CAFA3 dataset for both BPO and CCO.

### Fig C: Sequence identity distributions of the V162-V198 training and testing datasets.

The sequence identity distribution of the V162-V198 training and testing datasets is shown as a histogram with proportions calculated as the number of amino acid matches divided by sequence length of the query protein based on the top-scoring hit from BLASTp searches with default settings.

### Fig D: CrowdGO applied to functionally annotate the complete proteomes of taxonomically diverse species.

Results for eight species are shown in main text Figure 3, here results for the remaining four species are presented. CrowdGO consensus annotation results were compared with existing Gene Ontology (GO) term annotations from UniProt, and with the subsets of manually curated SwissProt (where available) and automatically inferred TrEMBL annotations, for (A) *Pan troglodytes* Chimpanzee and *Homo sapiens* human, (B) *Arabidopsis thaliana* the thale cress (repeated here for plant-plant model-non-model comparison) and *Solanum lycopersicum* Tomato, and (C) *Candidatus Thorarchaeota archaeon* SMTZ1-45. Bars at the top of each panel show the total numbers of proteins annotated with at least one GO term for each respective annotation dataset, with white-filled areas showing the remaining proteins with no annotations. Split violin plots show the distributions of the numbers of GO Slim terms annotated per protein (purple, left), and summed leaf+parents information content (IC) per protein (green, right). Y-axes are limited to a maximum of value of 300 for total protein IC distributions. The boxplots show the medians and 1.5 times the interquartile range for the numbers of GO Slim terms annotated per protein.


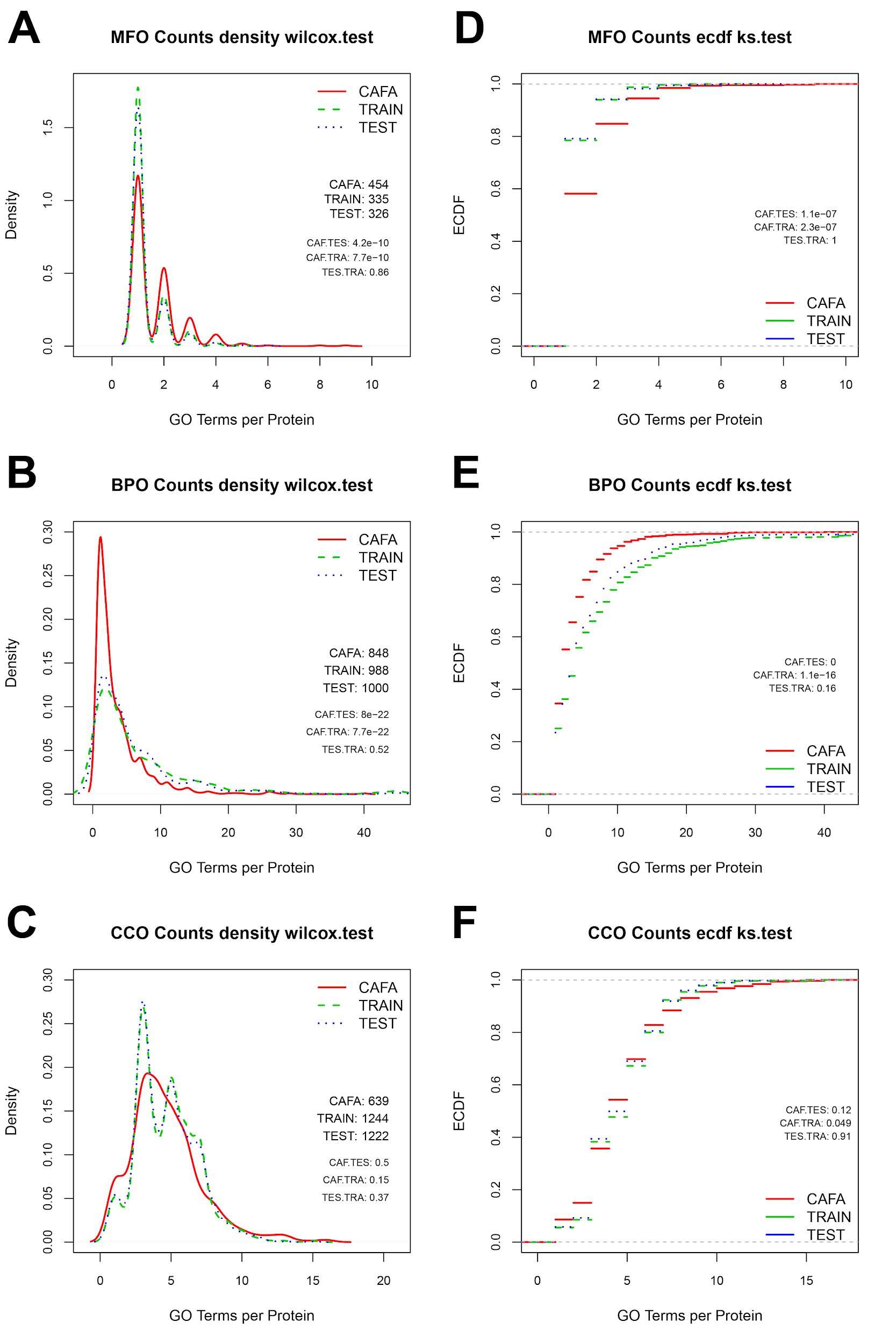


**Figure A**


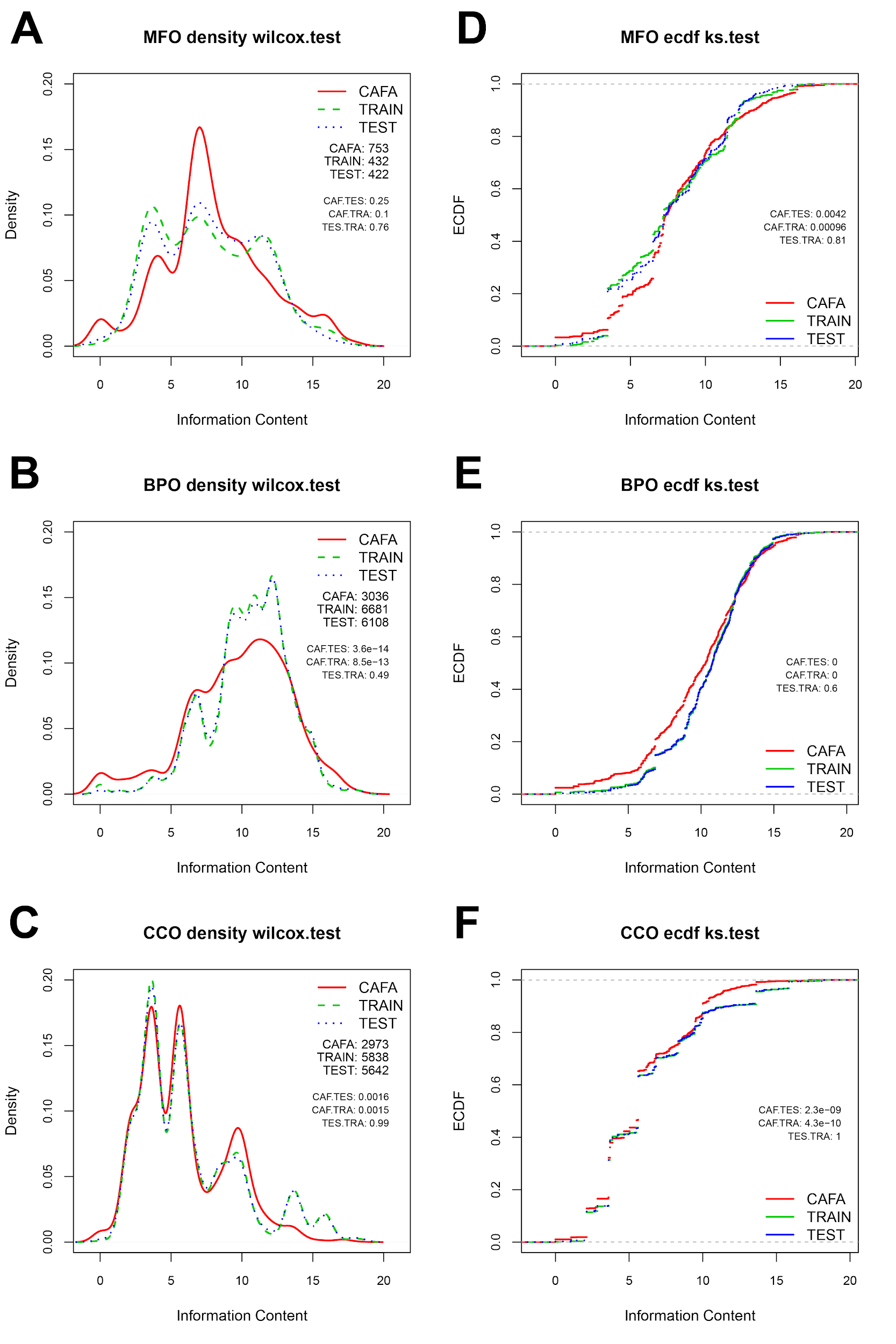


**Figure B**

**
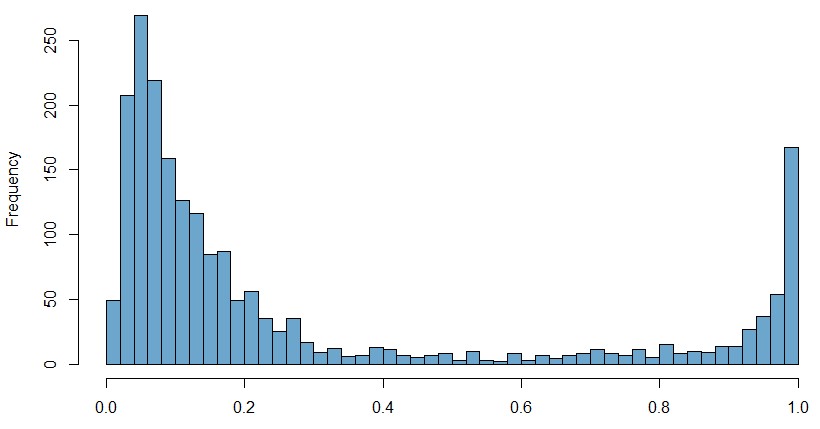
**

**Figure C**

**
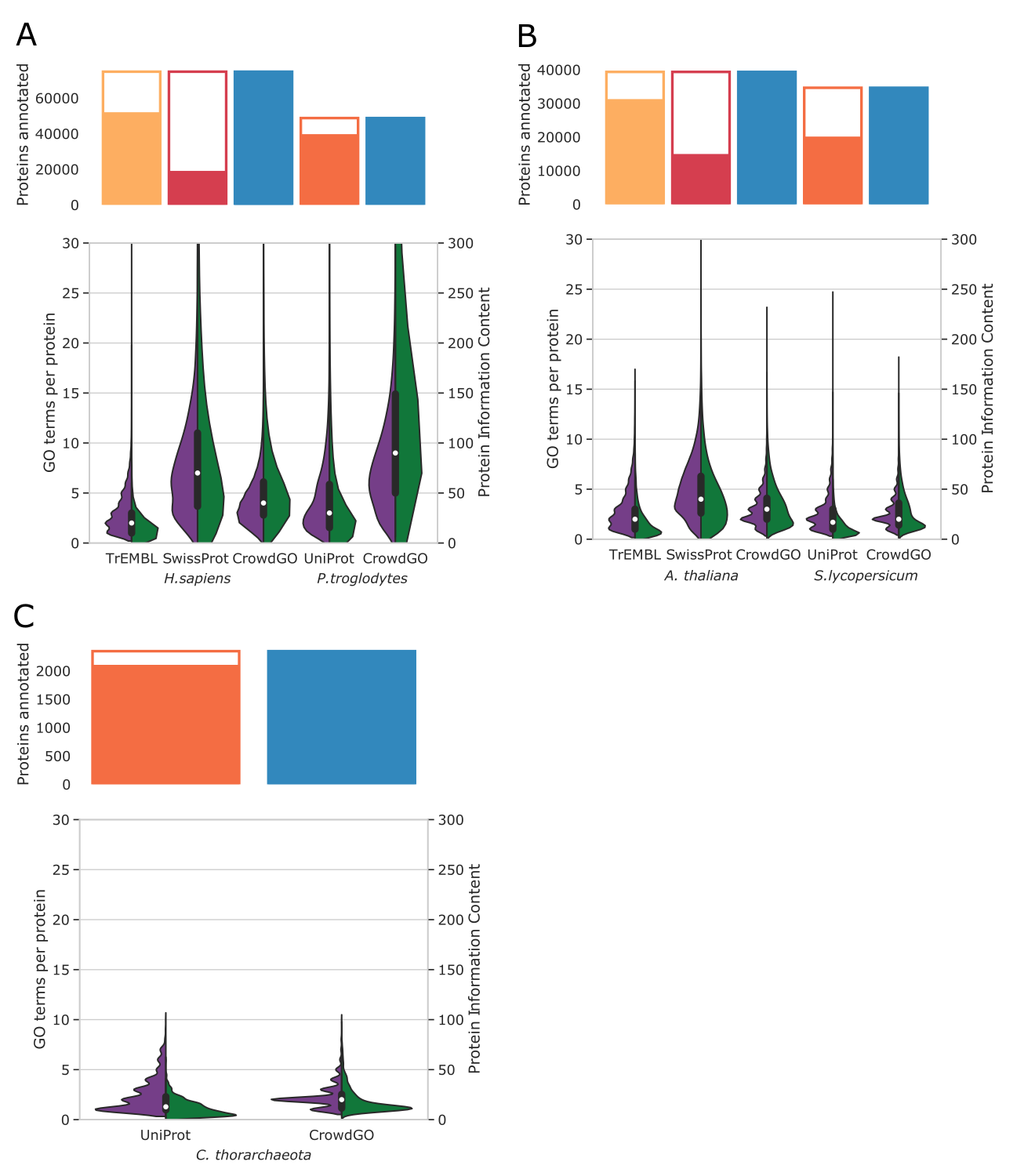
**

**Figure D**

# Supplementary References

1. Zhou N, Jiang Y, Bergquist TR, Lee AJ, Kacsoh BZ, Crocker AW, et al. The CAFA challenge reports improved protein function prediction and new functional annotations for hundreds of genes through experimental screens. Genome Biol. 2019;20: 244. doi:10.1186/s13059-019-1835-8

2. Kulmanov M, Hoehndorf R. DeepGOPlus: improved protein function prediction from sequence. Cowen L, editor. Bioinformatics. 2020;36: 422–429. doi:10.1093/bioinformatics/btz595

3. Reijnders MJMF. Wei2GO: weighted sequence similarity-based protein function prediction. PeerJ. 2022; 10:e12931. doi: 10.7717/peerj.12931

4. Jones P, Binns D, Chang H-Y, Fraser M, Li W, McAnulla C, et al. InterProScan 5: genome-scale protein function classification. Bioinformatics. 2014;30: 1236–1240. doi:10.1093/bioinformatics/btu031

5. Rentzsch R, Orengo C. Protein function prediction using domain families. BMC Bioinformatics. 2013;14: S5. doi: 10.1186/1471-2105-14-S3-S5

6. Buchfink B, Xie C, Huson DH. Fast and sensitive protein alignment using DIAMOND. Nat Methods. 2015;12: 59–60. doi:10.1038/nmeth.3176

7. Eddy SR. Accelerated Profile HMM Searches. Pearson WR, editor. PLoS Comput Biol. 2011;7: e1002195. doi:10.1371/journal.pcbi.1002195

8. El-Gebali S, Mistry J, Bateman A, Eddy SR, Luciani A, Potter SC, et al. The Pfam protein families database in 2019. Nucleic Acids Res. 2019;47: D427–D432. doi:10.1093/nar/gky995

9. Mi H, Poudel S, Muruganujan A, Casagrande JT, Thomas PD. PANTHER version 10: expanded protein families and functions, and analysis tools. Nucleic Acids Res. 2016;44: D336–D342. doi:10.1093/nar/gkv1194

10. Dawson NL, Lewis TE, Das S, Lees JG, Lee D, Ashford P, et al. CATH: an expanded resource to predict protein function through structure and sequence. Nucleic Acids Res. 2017;45: D289–D295. doi:10.1093/nar/gkw1098

11. Rentzsch R, Orengo CA. Protein function prediction using domain families. BMC Bioinformatics. 2013;14: S5. doi:10.1186/1471-2105-14-S3-S5

12. Friedberg I, Radivojac P. Community-Wide Evaluation of Computational Function Prediction. In: Dessimoz C, Škunca N, editors. The Gene Ontology Handbook. New York, NY: Springer New York; 2017. pp. 133–146. doi:10.1007/978-1-4939-3743-1_10

13. Clark WT, Radivojac P. Information-theoretic evaluation of predicted ontological annotations. Bioinformatics. 2013;29: i53–i61. doi:10.1093/bioinformatics/btt228
