# Supplementary material for: CrowdGO: Machine learning and semantic similarity guided consensus Gene Ontology annotation
Source: PLoS Comput Biol. 2022 May 13;18(5):e1010075. doi: 10.1371/journal.pcbi.1010075 (PMC9132264; doi:10.1371/journal.pcbi.1010075)
Supplement: Attachment — Submitted filename: PCOMPBIOL-D-21-01327_Comments-to-the-authors.pdf [file pcbi.1010075.s002.pdf]

The authors present CrowdGO, a consensus meta-predictor combining GO term predictions from 4 individual predictors. CrowdGO seems to improve over the predictions for the individual predictors and seems to be competitive to the top 3 CAFA3 competitors. However, the data generation process does not ensure non-redundancy between considered datasets which could lead to a bias and overestimation of performance. Also, some technical details need further clarification.

Major comments:

1. Dataset creation: While the authors ensure no overlap between training and testing, the CAFA3 benchmarks, and the training sets of the individual predictors, they only ensure that the same sequence is not present in 2 sets; however, similar sequences could still be present between the different sets. This could lead to a bias and overestimation of performance. To allow a fair performance assessment, non-redundancy between the various sets needs to be ensured.
    - Instead of just constructing the dataset from sequences added after 2017, those sequences also need to be redundancy reduced (common thresholds are 20-30% sequence identity) against (i) the CAFA3 benchmark sets and (ii) the 4 training sets of the 4 individual predictors.
    - In addition, the training set needs to be redundancy reduced against the test set to ensure that no protein in test is similar to another protein in the training set
    - To avoid a large decrease in proteins available for training/testing using this approach, the authors could consider using the entire data available in GOA and then redundancy reduce against the different training sets, the CAFA3 benchmarks and between the training and test split
2. How were the predictors chosen? The authors provide details for the selection process only in the section “Availability and Future Directions”; however, this crucial information should rather be moved to “Design and Implementation”. Also, while the authors justify not including the top 10 CAFA3 competitors, it is still unclear how they decided to use those particular 4 predictors given the vast number of available predictors including concepts different from the 4 methods used here and also methods published more recently
3. The authors state that 4 different models (CrowdGOFull, CrowdGOLight, CrowdGOFull-SwissProt, CrowdGOLight-SwissProt) were trained.
    - Why are those 4 different models needed?
    - In general, how does the performance for those 4 models differ? The authors only show results on the CAFA3 benchmark set.
    - According to Table 3, CrowdGOLight seems to perform identical to CrowdGOFull and is the less complicated model. Why is CrowdGOFull needed?
    - Which model is used when the authors refer to “CrowdGO”?
4. The authors should provide a more differential assessment of the performance of CrowdGO:
    - How would the performance look if only 2 or 3 of the 4 predictors were used?
    - How would the performance look if the 4 predictors were combined with e.g., a simple majority vote instead of training an AdaBoost?

5. Application to full proteomes: The authors state that CrowdGO increases the number of annotated proteins and the number of GO terms annotated to a certain protein. However, how many of those annotations could already have been obtained by e.g., using homology-based inference?
6. The authors should add error estimates (e.g., 95% confidence intervals as used in the CAFA assessment) to their performance comparisons (Tables 2, 3, Figure 1D). Are the performance differences statistically significant?

Minor comments:

1. Reference 7 (Reference 5 in the Supplement): This is not the right reference for the FunFams method to predict GO terms but the method presented in this paper uses FunFams to refine per-residue binding predictions.
2. P. 4: How was the threshold of 0.5 chosen? Is that the score leading to Fmax?
3. Table 3: Please add the names and references for the CAFA3 top3 methods
4. Table 3: How does the performance look for CAFA #4 – CAFA #10? Is the performance similar to the top 3 or is it clearly worse?
5. Application to full proteomes: The authors should add a statement about the speed. Can CrowdGO easily be applied to full proteomes? How long does it take to generate all predictions for e.g., human?
6. P. 14, l. 423-425: The authors state that CrowdGO "might benefit from developing additional models using eXtreme Gradient Boosting". Why did the authors not test that if they assume it could improve performance?
7. Why was the GO version from 2016 and not a more recent version used?
8. Running input predictors and CrowdGO: The described workflow is not really clear and should be rephrased. Were the predictors re-trained on the specified datasets or are those the sets used for the original training of the predictors?
9. CrowdGO software workflow: The authors could add a figure visualizing the software workflow which could be added to the main text.
10. Why were no other hyperparameters for the AdaBoost model tested?
