# Supplementary material for: CrowdGO: Machine learning and semantic similarity guided consensus Gene Ontology annotation
Source: PLoS Comput Biol. 2022 May 13;18(5):e1010075. doi: 10.1371/journal.pcbi.1010075 (PMC9132264; doi:10.1371/journal.pcbi.1010075)
Supplement: Attachment — Submitted filename: myreview.odt [file pcbi.1010075.s003.odt]

Summary/General Remarks

The manuscript describes a software tool that combines predicted functional annotations from different GO predictors using an ensemble method leading to improved performance. The authors benchmark their tool against the top-performing method from CAFA3 and report competitive performance for biological process and cellular component and slightly worse performance for molecular function predictions.
The code for tool is available with an open source license and contains a detailed README file with instructions on how to install and run the software and its dependencies. Currently, a few GO predictors are implemented and integrated, but it is straightforward for users to include more predictors. The tool could be of use to biologists who are interested in annotating new sequences as well as protein function prediction researchers as it provides a relatively strong baseline to compare new methods against.

Specific comments to the authors

Line 100: The input consists of predicted GO annotations from different methods in the form of posterior probabilities. Do these probabilities need to be consistent with the GO DAG (i.e. P(ancestor) >= P(descendant) )? Or does crowdGO internally make this correction?
Similar question for the output of crowdGO: Are the predictions guaranteed to respect the DAG hierarchy and if not, are they post-processed to make them so?
If someone wants to incorporate an additional predictor to a trained crowdGO model, does that imply that the adaboost algorithm has to be retrained from scratch?
Could the authors comment on the training time of the adaboost algorithm and its relation to the number of predictors used? Does it take considerably longer to train a model that combines more predictors?
The currently implemented predictors are all sequence-based, but for some model species, one might want to incorporate other data sources such as gene expression or protein interaction data, which are not available for most non-model species. Does crowdGO support “missing predictors” or would I need a separately trained model?
