# Supplementary material for: CrowdGO: Machine learning and semantic similarity guided consensus Gene Ontology annotation
Source: PLoS Comput Biol. 2022 May 13;18(5):e1010075. doi: 10.1371/journal.pcbi.1010075 (PMC9132264; doi:10.1371/journal.pcbi.1010075)
Supplement: Attachment — Submitted filename: Response to reviewers.docx [file pcbi.1010075.s004.docx]

# Reviewer #1's Responses to Questions

Comments to the Authors:

Please note here if the review is uploaded as an attachment.

Reviewer #1: The review is uploaded as an attachment.

The authors present CrowdGO, a consensus meta-predictor combining GO term predictions from 4 individual predictors. CrowdGO seems to improve over the predictions for the individual predictors and seems to be competitive to the top 3 CAFA3 competitors. However, the data generation process does not ensure nonredundancy between considered datasets which could lead to a bias and overestimation of performance. Also, some technical details need further clarification.

Response: We respond to the issue on sequence redundancy below, and we provide the requested clarifications on the technical details.

## Major comments:

1. Dataset creation: While the authors ensure no overlap between training and testing, the CAFA3 benchmarks, and the training sets of the individual predictors, they only ensure that the same sequence is not present in 2 sets; however, similar sequences could still be present between the different sets. This could lead to a bias and overestimation of performance. To allow a fair performance assessment, non-redundancy between the various sets needs to be ensured.

Response: We agree with the reviewer that careful consideration of the needs for sequence redundancy reduction is important when building datasets for assessing the performance of classifiers. This would be particularly important when assessing the performance of methods that are specifically designed to be homology-independent, e.g. FFPred. In order to conduct like-for-like comparisons using the CAFA3 benchmark dataset and our V162-V198 training and testing datasets we followed the CAFA3 dataset generation guidelines explicitly, which do not include sequence redundancy reduction. This reflects the real-world scenario faced when attempting to annotate functions of new proteins from new species where redundancy (similar sequences) and uniqueness are both expected. This is particularly relevant for all methods that rely on or use sequence similarity as the reality is a spectrum of similarities where a key consideration is where to draw the line (cut-off) to achieve the best results. To ensure that this caveat is not leading to bias and overestimation of performance we have now assessed the sequence identity distribution of the V162-V198 training and testing datasets (new Figure S3). The distribution follows a pattern very similar to those shown previously for the CAFA datasets. Therefore, in accordance with CAFA3 guidelines, we maintain the use of non-redundancy-reduced datasets for the comparisons of the consensus CrowdGO results with those from each of the four input predictors, and with the top-performing methods from CAFA3. We do however acknowledge the importance of considering the effects of redundancy reduction, and we have therefore made sure to clearly state that this step was not part of the dataset generation procedure that we employed.

o Instead of just constructing the dataset from sequences added after 2017, those sequences also need to be redundancy reduced (common thresholds are 20-30% sequence identity) against (i) the CAFA3 benchmark sets and (ii) the 4 training sets of the 4 individual predictors.

Response: See above.

o In addition, the training set needs to be redundancy reduced against the test set to ensure that no protein in test is similar to another protein in the training set

Response: See above.

o To avoid a large decrease in proteins available for training/testing using this approach, the authors could consider using the entire data available in GOA and then redundancy reduce against the different training sets, the CAFA3 benchmarks and between the training and test split

Response: See above. The aim was to follow community guidelines on dataset generation.

2. How were the predictors chosen? The authors provide details for the selection process only in the section “Availability and Future Directions”; however, this crucial information should rather be moved to “Design and Implementation”.

Response: We would have liked to include more/other tools, however, in order to perform the training and testing as described we need open access tools with annotation database versions that can be altered/selected to match exactly the date-based requirements for building the correct datasets that do not overlap in content. For most of the available, well-performing tools, such as for example the ‘winner’ of CAFA3 (NetGO / GOLabeler) it is not possible to alter the database versions to correspond exactly to the required conditions for like-for-like training and testing dataset generation. Without the ability to select datasets from fixed-time-built databases we cannot train a CrowdGO model that incorporates such tools. This is because tool-specific training datasets cannot be allowed to overlap with our testing datasets (the CAFA3 benchmarking and the V162-V198 testing datasets). To include any other method would require this method to be trained using only annotations acquired prior to the generation of the testing datasets. Thus where we have no control over the versioning of the training datasets used, e.g. with web server-only tools like Argot2.5, we are unable to use them to build CrowdGO models that can be properly assessed for performance using standard community procedures. The predictors that were chosen were therefore based on the fact that we could build the version-controlled databases required for like-for-like fair comparisons. Importantly, it should be noted that users can still easily train their own CrowdGO models using their favourite predictors - but here for demonstrating performance we could only use predictors for which we could control the database versions. The issue of the need for fully open access to tools and the ability to re-train with user-defined datasets is a problem that the field as a whole is acknowledging but so far finding difficult to solve across all types of methods new and old. As suggested by the reviewer, for clarity we have now mentioned this also in the “Design and Implementation” section.

Also, while the authors justify not including the top 10 CAFA3 competitors, it is still unclear how they decided to use those particular 4 predictors given the vast number of available predictors including concepts different from the 4 methods used here and also methods published more recently

Response: As detailed above, the selection here is mainly practical as we can only properly assess individual input predictors for which we can control/select the appropriate training datasets. Other models can be built that incorporate results from other methods, and CrowdGO provides users with the means to do this, however objective testing of performance is not possible when the training data for each method cannot be clearly shown to be non-overlapping with any testing datasets.

3. The authors state that 4 different models (CrowdGOFull, CrowdGOLight, CrowdGOFull-SwissProt, CrowdGOLight-SwissProt) were trained.

o Why are those 4 different models needed?

Response: We agree that this was not made entirely clear in the text and have now added a brief explanation. We built four different models for CrowdGO that are designed to cater to different users’ needs and/or available computational storage and/or compute power. The most comprehensive model requires users to install and run all four predictor methods, and to use the entire TrEMBL database of proteins. The other models reduce this burden with the ‘Light’ models that exclude InterProScan - for which installation can be challenging in different compute environments and for which annotations can have long runtimes. The ‘SwissProt-only’ models remove the burden of having to use the entire TrEMBL database (51 gigabyte zipped) and instead work with the much smaller SwissProt database (86 megabyte zipped).

o In general, how does the performance for those 4 models differ? The authors only show results on the CAFA3 benchmark set.

Response: We apologise for this omission, we had planned to show the results of the other three CrowdGO models alongside CrowdGOFull in Table 2. This has now been updated so that the performance of the no-InterPro and SwissProt-only models can be compared with the full model. Compared to the CrowdGOFull model, the ‘Light’ and ‘SwissProt-only’ models show overall slightly lower Fmax and AUPR scores, together with slightly higher Smin values, indicating that the CrowdGOFull does produce the best results, while the ‘Light’ and ‘SwissProt-only’ models still perform better than the individual input predictors by almost all measures.

o According to Table 3, CrowdGOLight seems to perform identical to CrowdGOFull and is the less complicated model. Why is CrowdGOFull needed?

Response: The differences amongst the performances of the four models based on the CAFA3 benchmarking dataset as shown in Table 3 do indeed appear small with little-to-no added benefit of using the CrowdGOFull model. However, comparing their relative performances on the much larger and up-to-date V162-V198 datasets shows the overall higher Fmax and AUPR scores and lower Smin values that can be achieved using the CrowdGOFull model. On the older, smaller CAFA3 dataset there appears to be no difference between using InterProScan results or not, this likely reflects that the InterPro2GO mappings were not as comprehensive as they are now.

o Which model is used when the authors refer to “CrowdGO”?

Response: The default model is the CrowdGOFull model, we have made sure now to specify this throughout the text wherever there could have been any ambiguities regarding the model we used.

4. The authors should provide a more differential assessment of the performance of CrowdGO:

o How would the performance look if only 2 or 3 of the 4 predictors were used?

Response: We have shown this only for model variations that exclude one predictor (CrowdGOLight) or models that use a smaller reference database for one predictor (-SwissProt models). This was mainly for practical considerations for the users, but it does demonstrate differences in terms of reduced performances. These are understandably minor when considering the lower overall added value provided by InterProScan as seen in Figures 1D and 2D,E,F. CrowdGO is designed as a meta-predictor, we would therefore not recommend reducing the number of input predictors to results from just two methods. It is likely that most users will use one of the provided models with 3 or 4 predictors, for more advanced users other models can be built that incorporate results from other methods and CrowdGO provides users with the means to do this, which we have emphasised now in the revised text.

o How would the performance look if the 4 predictors were combined with e.g., a simple majority vote instead of training an AdaBoost?

Response: Given the numbers of false positives shown in figures 2D,E,F it is clear that a simple majority vote system would result in the calling of many false positives. The trained models learn how to re-score annotations so low-confidence annotations coming from the majority of methods would still be rejected while high-confidence annotations coming from a single method would still be accepted. This concept was briefly mentioned in the “Availability and Future Directions” section.

5. Application to full proteomes: The authors state that CrowdGO increases the number of annotated proteins and the number of GO terms annotated to a certain protein. However, how many of those annotations could already have been obtained by e.g., using homology-based inference?

Response: Here we aim to show that by applying CrowdGO to annotating full proteomes users can obtain annotation sets with increased numbers of annotated proteins and GO terms annotated to a certain protein compared to simply sourcing existing annotations from SwissProt, UniProt, or TrEMBL. It is true that many of these annotations could also be obtained by applying homology-based inference methods, but as shown in Figure 2 such methods can produce many false positives. Therefore the advantage of the CrowdGO consensus approach is that it results in more confident annotations without reducing the overall coverage of annotations compared with those that can be sourced from standard community databases. We have added this important aspect to the text to clarify this for the readers.

6. The authors should add error estimates (e.g., 95% confidence intervals as used in the CAFA assessment) to their performance comparisons (Tables 2, 3, Figure 1D). Are the performance differences statistically significant?

Response: Thank you for the suggestion, we have now added error estimates to the barchart in Figure 1D where they help to visually distinguish true differences in the Fmax scores. For the CAFA3 comparisons, while confidence intervals are presented in the figures of Zhou et al. the actual values are not reported therefore we cannot add them to the table.

## Minor comments:

1. Reference 7 (Reference 5 in the Supplement): This is not the right reference for the FunFams method to predict GO terms but the method presented in this paper uses FunFams to refine per-residue binding predictions.

Response: Thank you for noting our erroneous citation, we have corrected this in both the main text and the supplement.

2. P. 4: How was the threshold of 0.5 chosen? Is that the score leading to Fmax?

Response: The threshold of 0.5 is the default value from AdaBoost, for simplicity and coherence across the three ontologies we therefore selected this value to apply as the standard cutoff. It is also in each case very close to the threshold values that correspond to the Fmax: 0.495 for CC, 0.51 for MF, 0.4768 for BP - these exact values have now been added to the manuscript text for clarity. As CrowdGO presents a much smoother relationship between precision and recall across thresholds, users may select different thresholds according to their needs to either focus on only the most reliable highest scoring annotations or to include the lower scoring annotations that steadily increase recall while reducing reliability.

3. Table 3: Please add the names and references for the CAFA3 top3 methods

Response: We have now added the names and references for the CAFA3 top3 methods in Table 3.

4. Table 3: How does the performance look for CAFA #4 – CAFA #10? Is the performance similar to the top 3 or is it clearly worse?

Response: We have added a row to Table 3 presenting the Fmax ranges for methods #4 to #10 for each ontology.

5. Application to full proteomes: The authors should add a statement about the speed. Can CrowdGO easily be applied to full proteomes? How long does it take to generate all predictions for e.g., human?

Response: We refrained from commenting on runtimes because this depends very much on the compute infrastructures available to users and the flexibilities of each of the input predictors. We have nevertheless provided an indication of expected times by adding some information about runtimes to the supplementary materials, indicating that the longest step is running InterProScan and the fastest step is actually using CrowdGO to produce the consensus annotations.

6. P. 14, l. 423-425: The authors state that CrowdGO “might benefit from developing additional models using eXtreme Gradient Boosting”. Why did the authors not test that if they assume it could improve performance?

Response: This statement is presented in the section “Availability and Future Directions”, because it is something that could be worth considering exploring in the future. We are not assuming that eXtreme Gradient Boosting would improve performance, simply noting that discussions in the field seem to indicate that in some circumstances eXtreme Gradient Boosting can out-perform AdaBoost. We are happy to remove this statement if needed.

7. Why was the GO version from 2016 and not a more recent version used?

Response: We used the GO version that matches the versions of the annotations because using a newer GO version would result in mismatches where terms have been deprecated or replaced with updated identifiers and refined definitions.

8. Running input predictors and CrowdGO: The described workflow is not really clear and should be rephrased. Were the predictors re-trained on the specified datasets or are those the sets used for the original training of the predictors?

Response: The individual input predictors were not re-trained, they were used as provided with their corresponding datasets that pre-date the annotations in the testing dataset. E.g. from the supplement: “DeepGOPlus (GitHub 13-09-2020, using the DeepGOPlus 2016 dataset) was run with default parameters.”; “Wei2GO v1.0 was run with default parameters using sequences and annotations from UniProt release 2016-11 and profiles from Pfam-A [8] version 30 (2016-05), i.e. with datasets that predate the sequences in the V162-V198 dataset to ensure no overlaps. InterProScan version 5.21.60 (2016-11) was run with default parameters and the --goterms option to obtain GO term annotations, using all default member databases as well as PANTHER protein families from version 10 [9], i.e. families that predate the sequences in the V162-V198 dataset. FunFams (cath-tools-genomescan GitHub 17-12-2019) was run with default parameters with CATH database version 4.1 [10], which was based on Protein Data Bank (PDB) release 01-01-2015.” We have rephrased the introductory sentences to clarify this.

9. CrowdGO software workflow: The authors could add a figure visualizing the software workflow which could be added to the main text.

Response: In lieu of an additional figure we have added a summary overview at the start of the description of the CrowdGO software workflow.

10. Why were no other hyperparameters for the AdaBoost model tested?

Response: Formal optimisation of hyperparameters was not carried out, just exploratory analyses.

# Reviewer #2's Responses to Questions

The manuscript describes a software tool that combines predicted functional annotations from different GO predictors using an ensemble method leading to improved performance. The authors benchmark their tool against the top-performing method from CAFA3 and report competitive performance for biological process and cellular component and slightly worse performance for molecular function predictions.

The code is available with an open source license and contains a detailed README file with instructions on how to install and run the software and its dependencies. Currently, a few GO predictors are implemented and integrated, but it is straightforward for users to include more predictors. The tool could be of use to biologists who are interested in annotating new sequences as well as protein function prediction researchers as it provides a relatively strong baseline to compare new methods against.

Response: We thank Reviewer #2 for their positive evaluations and recognition of the benefits that CrowdGO will bring to the field of protein function prediction.

## Specific comments to the authors

1. Line 100: The input consists of predicted GO annotations from different methods in the form of posterior probabilities. Do these probabilities need to be consistent with the GO DAG (i.e. P(ancestor) >= P(descendant) )? Or does crowdGO internally make this correction?

Response: Yes the results are consistent with the DAG. Where used, propagating up the DAG makes sure that a parent term has the same or greater probability than the child term. Manual propagation is not performed during input preparation for CrowdGO, but where applicable during internal calculations of CrowdGO, i.e. preparing the features for the AdaBoost model, the DAG is respected so that an ancestor cannot have a lower probability than a descendant. Additionally, DeepGOPlus produces output which includes probabilities all along the DAG, respecting this same rule.

2. Similar question for the output of crowdGO: Are the predictions guaranteed to respect the DAG hierarchy and if not, are they post-processed to make them so?

Response: Yes the predictions are guaranteed to respect the DAG hierarchy. Output of CrowdGO respects the DAG so that the probability of an ancestor cannot be smaller than the probability of a descendant. For the evaluation of CrowdGO in the manuscript, manual propagation was done respecting this same rule.

3. If someone wants to incorporate an additional predictor to a trained crowdGO model, does that imply that the adaboost algorithm has to be retrained from scratch?

Response: Yes, users may train their own new models incorporating additional predictors using the accessory functions provided with CrowdGO. This is now explicitly highlighted in the revised text, along with the instructions on the GitLab repository.

4. Could the authors comment on the training time of the adaboost algorithm and its relation to the number of predictors used? Does it take considerably longer to train a model that combines more predictors?

Response: The decision tree complexity is O(nkd) with n = samples, k = features, and d = depth.The weak learner used by AdaBoost is a Decision Tree with a fixed depth of 2, with 10.000 iterations. Hence, adding features would increase the runtime linearly. However, adding an input predictor does not increase the amount of features linearly, as some features are the results of all-vs-all semantic similarities between GO terms of the different input predictors, i.e. the 4 predictors used for the CrowdGOFull model produce 6 all-vs-all features, and adding an additional predictor would produce 10 all-vs-all features. As a result, adding one or two predictors would increase the time needed for training close to linear, but adding a hypothetical 20 extra predictors would take considerably more time.

5. The currently implemented predictors are all sequence-based, but for some model species, one might want to incorporate other data sources such as gene expression or protein interaction data, which are not available for most non-model species. Does crowdGO support “missing predictors” or would I need a separately trained model?

Response: A separately trained model would need to be built. If a particular predictor that uses other sources such as gene expression or protein interaction data can be applied to the species in question, then a CrowdGO model that incorporates these can be built. So in this sense ‘missing predictors’ are not supported, i.e. producing CrowdGO consensus annotations from results from predictors 1, 2, 3, and 4 would require a model trained on results from predictors 1, 2, 3, and 4. We have clarified this in the main text.
