# Supplementary material for: CrowdGO: Machine learning and semantic similarity guided consensus Gene Ontology annotation
Source: PLoS Comput Biol. 2022 May 13;18(5):e1010075. doi: 10.1371/journal.pcbi.1010075 (PMC9132264; doi:10.1371/journal.pcbi.1010075)
Supplement: Attachment — Submitted filename: Response to reviewers2.docx [file pcbi.1010075.s005.docx]

Dear Editors,

Thank you for your guidance with respect to the remaining necessary responses. We have now performed the additional analyses to compare CrowdGO annotations with a homology-based inference approach, as detailed below. We note also that we updated the reference for Wei2GO as it was previously a pre-print at bioRxiv and has now been published at PeerJ.

Yours faithfully,

Maarten Reijnders and Robert Waterhouse

Reviewer 1 continues to have comments on the revised manuscripts.  You have responded adequately to Reviewer 1's comments, with one exception which we'd like to determine if you can better address.

**Reviewer 1 makes the following request based on the revised manuscript:**

**4. Application to full proteomes**: I agree that just applying homology-based inference without any threshold will result in many false positive. However, how many of the annotations from CrowdGO could be obtained through homology-based inference using a common E-value threshold or similar? Again, I understand why applying CrowdGO is (probably) superior to applying homology-based inference. However, if the authors believe that why not support it with the respective results?

Please consider if CrowdGO's improvement can actually be quantified to demonstrate clearly how much better that CrowdGO was from simple homology modeling.  Otherwise, the manuscript is acceptable and does not need to respond to Reviewer 1's remaining comments on this revision.

To address the question of how many CrowdGO annotations could be obtained through simple homology-based inference we annotated the 12 proteomes using an approach similar to the CAFA3 baseline. These enabled comparisons that showed that annotations from applying CrowdGO overall outnumbered those obtained using homology-based inferences (but slightly fewer for BPO). Only 53% MFO, 44% BPO, and 39% CCO of CrowdGO annotations were also predicted by the baseline approach. We have added this to the main text, and outlined the methodology and full results in the supplementary materials (including a new table, Table S2).
